# What does safety in mental healthcare transitions mean for service users and other stakeholder groups: An open‐ended questionnaire study

**DOI:** 10.1111/hex.13190

**Published:** 2021-01-20

**Authors:** Natasha Tyler, Nicola Wright, Maria Panagioti, Andrew Grundy, Justin Waring

**Affiliations:** ^1^ Faculty of Biology, Medicine and Health NIHR Greater Manchester Patient Safety Translational Research Centre (PSTRC) University of Manchester Manchester UK; ^2^ School of Health Sciences University of Nottingham Nottingham UK; ^3^ NIHR Greater Manchester Patients Safety Translational Research Centre University of Manchester Manchester UK; ^4^ University of Nottingham Nottingham UK; ^5^ Health Services Management Centre University of Birmingham Birmingham UK

**Keywords:** care transitions, discharge, mental health, patient safety, safety, thematic analysis

## Abstract

**Background:**

Historically, safety mental health research has tended to focus on risks of homicide, suicide and deaths. Although wider safety issues are now recognized in regards to mental health services, the safety of mental health transitions, a key research and policy priority according to World Health Organisation, has not been explored.

**Objective:**

The purpose of this study was to investigate perceptions of safety in mental health transitions (hospital to community) amongst five stakeholder groups.

**Design and setting:**

An online, international cross‐sectional, open‐ended questionnaire.

**Participants:**

There were five stakeholder participant groups: service users; families/carers; mental health‐care professionals; researchers; and end users of research.

**Results:**

Ninety‐three participants from 12 different countries responded. Three overarching themes emerged: ‘individual/clinical’, ‘systems/services’ and ‘human, behavioural and social’ elements of safe mental health transitions. Whilst there was a great focus on clinical elements from researchers and healthcare professionals, service users and carers considered safety in terms of human, behavioural and social elements of transitional safety (ie loneliness, emotional readiness for discharge) and systems/services (ie inter‐professional communication).

**Discussion:**

Safety in mental health‐care transitions is perceived differently by service users and families compared to healthcare professionals and researchers. Traditional safety indicators for care transitions such as suicide, self‐harm and risk of adverse drug events are raised as important. However, service users and families in particular have a much wider perception of transitions safety.

**Conclusion:**

Future quality and safety research and policy should consider including a service user voice and consider integration of psychosocial elements in discharge interventions.

## 1 INTRODUCTION

Historically, the safety focus in mental health research and practice has tended to focus on the risks of homicide, suicide and deaths.^1^ More recently, wider safety issues have been described and classified in regards to the organization of mental health services.^2^ A recent paper considered the safety issues that span inpatient and community mental health care.^2^ The researchers attempted to apply an existing framework for patient safety incident investigation, the Yorkshire Contributory Factors Framework (YCFF), to a mental health context. However, they found that service user and professional reported safety issues did not map directly onto the YCFF, which necessitated the addition of two further categories ‘social environment’ and ‘service process’. The latter primarily concerned care transitions and access to services. Although there has been increased attention to the safety of care transitions in other care services,^3^ there is comparatively little research on the safety issues associated with the transition from acute to community mental health services.

Care transitions such as discharge from mental health hospitals to the community have been described as an especially risky phase.^4^ The World Health Organisation's (WHO) global strategy for patient safety specifically targets care transitions as a basis for reducing patient harm and improving patient safety.^5^ Multiple systems‐level risk factors for safety have been found in mental health‐care transitions, namely the lack of continuity of care, co‐ordination and communication difficulties between organizations and professionals.^6^, ^7^, ^8^, ^9^ Various interventions have been developed to improve safety in mental health services^10^, ^11^, ^12^, ^13^, ^14^ but very few explicitly describe an underlying theory of change.^15^ Yet it is increasingly recognized that the evaluation of quality improvement interventions, such as those for hospital discharge, should more overtly articulate and appraise the underpinning theory of change for a given intervention (the rationale and assumptions about mechanisms that links processes and inputs to outcomes, also specifying the conditions necessary for effectiveness).^16^, ^17^ In order to develop a theory of change that underpins effective interventions, researchers need to understand what they are looking to address (ie which specific safety issues) and determine how improvement of these safety issues will be captured, measured or reported. This study aims to understand what the safety issues are for each group, to enable future intervention developers to implement theory of change more readily.

Working closely with experts by lived experience in mental health research is thought to be beneficial for both service users and researchers.^18^ Mental health is one area of research and practice that is thought to particularly benefit from increased involvement and ensuring the service user voice is heard.^18^, ^19^ In order to evidence the importance of service user involvement in mental health quality and safety research, understanding how service users and carers perceive safety differently is imperative. Articulating and reporting specifically how views of quality and safety in mental health‐care transitions differ between stakeholder groups (service users, families and carers, mental health professionals, policy makers/end users of research and international researchers), not only translates into meaningful user‐relevant research, but can also provide a framework for translation into meaningful changes in practice and enable a widening of traditional safety definitions. Wider definitions could facilitate the development of relevant ‘theory of change’ to underpin future quality and safety interventions that have potential to improve safety by all stakeholder definitions.

## 2 OBJECTIVE

The purpose of this study was to investigate perceptions of safety outcomes and priorities in mental health transitions (hospital discharge to the community) amongst five stakeholder groups including service users and families/carers, as well as mental health‐care professionals, researchers and end users of research. We also wanted to determine whether service users and their families/carers would raise different safety outcomes and priorities in mental health transitions than health‐care professionals, researchers and end users of research. We anticipated that service users and their families/carers would perceive safe discharge more widely incorporating social, behavioural and human elements in their responses whereas health‐care professionals, researchers and end users of research would focus primarily on clinical or traditionally measured outcomes.

## 3 METHODS

This paper involved secondary analysis of data collected to develop a core outcome set for research into discharge from acute mental health services.^20^ We present an interpretative qualitative analysis of data gathered during an online open‐ended questionnaire that was synthesized into outcomes for previous research. As so many individuals took time to share their perspectives of safety in care transitions we did not want to misrepresent the meaningful, elaborate responses received by distiling them only into potential outcomes and not exploring them thematically.

### 3.1 Participants

Participants were recruited to an online consensus panel to develop a core outcome set for mental health discharge.^20^ We used an opportunistic sample to gather data from five stakeholder groups: individuals with lived experience (service users), families and carers, researchers, healthcare professionals (HCPs) and end users of research (the people who will use research to make decisions—ie policy makers, service managers, charity workers and commissioners).

International researchers were invited to participate whether they had published a peer‐reviewed paper in a previous systematic review, 50 researchers were invited.^15^ Twenty‐nine British end users of research were identified using publicly accessible contact details and individually invited to participate. Service users, HCPs and end users of research were recruited via adverts on social media (Twitter and Instagram). Every participant who agreed to be involved completed the questionnaire. There were no restrictions on country of origin for social media respondents and responses were quality assessed by one researcher (NT) to ensure these participants had relevant knowledge about the topic. The researcher has experience of ethnographic observations of discharge on mental health wards and any concerns were discussed with a qualified mental health professional on the team (NW).

### 3.2 Materials

The questionnaire was administered on a secure online platform, Qualtrics.^21^ The questionnaire assessed attitudes to safety and outcomes in mental health‐care transitions. All questions elicited free‐text responses. This included four open‐ended items that were presented to all participants and specific open‐ended questions for each stakeholder group (four for service users and two for all other groups; Table S1). The four questions asked to all focused on (a) what makes discharge safe? (b) what makes discharge effective? (c) future research priorities and (d) outcome measures.

The questions were loosely modelled on a questionnaire developed for a large‐scale outcome generation study for a depression core outcome set that were developed with service users and healthcare professionals.^22^ The question formation was mirrored but adapted for a mental health discharge theme. For example, ‘For you, what is the most difficult aspect of depression to live with or endure?’ was edited to ‘What do you think is the most difficult aspect of discharge from a mental health acute ward?’. The views of a patient and public involvement (PPI) group were sought to confirm the appropriateness of questions and instructions and to pilot the questionnaire (n = 5).

### 3.3 Design

A cross‐sectional design was used.

### 3.4 Procedure

Participants completed the questionnaire between December 2018 and January 2019. Participants either received an email link or followed a social media link. After giving consent, they were presented with the questionnaire then invited to record their email address for follow‐up for the core outcome set study.^20^ The email addresses were stored separately from each participant's data and used only for follow‐up contacts.

### 3.5 Analysis

We collected basic, descriptive data around participant demographics (age, gender, stakeholder group and country of residence). The qualitative data collected from the questionnaire were analysed thematically using conventional qualitative techniques proposed by Ritchie and Spencer.^23^ This involved coding the individual participant responses and then grouping these together as ‘meaning units’. These grouped units were then assigned consolidated codes, and the similarities and differences between them were compared. A further consolidation process led to the development of overarching themes to explain the data. The initial and majority of the analysis was conducted by NT. The themes and analysis were then discussed within the wider research team for verification purposes (NW, JW, MP and AG). The team discussed and agreed upon what the key distinctions were between codes and themes. If a theme could be described in a way that particularly highlighted differences between the groups, this would take precedent over other potential theme descriptions. For example, ‘readiness for discharge’ could also be thematically captured as ‘discharge planning’; however, ‘readiness for discharge’ highlights the difference in opinions between groups as for service users and family/carers this encapsulated much more than the clinical process of planning discharge. The aim of the discussion with the wider group was to increase trustworthiness and credibility of the data, by generating to consensus amongst five academics who also represented many of the sampled groups (a clinical academic, a peer researcher (service user) and two family members of service users).

### 3.6 Patient and public involvement

A PPI group (5 members) was involved throughout the design and conduct of this research. For example, online questionnaires were appraised by this group, who helped provide feedback and suggest changes to the content from a lay perspective. An expert by lived experience (peer researcher) was part of the study team and present at each research meeting, contributing to the design, conduct, analysis and manuscript preparation.

### 3.7 Ethics

This study was approved by the ethics committee at the University of Nottingham Business School. Informed consent was gained from all participants. Participants were made aware that they could withdraw from the study at any time.

## 4 RESULTS

### 4.1 Participants

Ninety‐three participants from 12 countries completed the questionnaire. Twenty‐seven identified as service users, 17 family/carers, 39 health‐care professionals, 15 end user of research and 37 researchers; however, many chose multiple categories. Table S2 shows the participant demographics.

### 4.2 Responses

The qualitative data highlighted three overarching themes in relation to issues of safety at care transition points in mental health: clinical, service level and human, behavioural and social. Table 1 summarizes the relationship between the overarching and identified subthemes within.

**TABLE 1:** Overarching themes and subthemes from the thematic analysis

Overarching theme	Subthemes
Individual/clinical	Suicide, self‐harm, violence and risk Medication management Symptoms/mental health
Services/systems	Readmission Discharge planning Integration and communication between services
Human, behavioural and social	Readiness for discharge Social networks and support Adaptation to normal life Knowledge Isolation/Loneliness

### 4.3 Individual/Clinical

The responses concerning the clinical elements of safety in mental health discharge had three main subthemes: (a) suicide, self‐harm, violence and risk, (b) medication management and (c) symptoms/mental health.

### 4.4 Suicide, self‐harm, violence and risk

Many of the researchers and HCPs in the sample described the importance of measuring/assessing suicide, self‐harm and violence during discharge from acute mental health services. The language used by many respondents in these groups indicated discharge is only effective when there are no self‐harm events, often described as a list of objectives to avoid to indicate an effective discharge *‘Reduction of suicide risk, No self‐harm’* (researcher). The idea that suicide, self‐harm and violence are the most important safety indicators was exemplified in many of the responses concerning what makes a safe discharge.

Making sure that the patient does not harm themselves or others (suicide, homicide, self‐mutilation, aggression)—researcher.

Risk and safety were often synonymous in the responses of HCPs, whereby *‘no risk’* typified safety at discharge. Researchers also described the importance of managing and assessing risk using standardized measures or processes, for example *‘robust risk assessment’* (mental health nurse) or *‘managed risk of suicide’* (researcher). Despite the general trend for professional groups to describe safety primarily in terms of suicide, self‐harm, violence and risk, some acknowledged the importance of taking a wider approach to other neglected elements of safety.

While safety and risk management are essential, it would be good to see positive aspects of discharge being prioritised as well ‐ directions for recovery, personal growth, education, improved health and opportunities to build support networks, which could serve as protective factors and help to prevent readmission.—clinical psychologist.

A contrasting response was received by service users and families within this subtheme. Only one service user described *‘risk’*, but they were also a healthcare professional. No service users or family/carers described suicide, self‐harm or violence in relation to safety at discharge.

### 4.5 Medication management

A similar polarization of responses was seen with other clinical elements of safety, only one service user described medication. Contrastingly, researchers and HCPs considered this imperative. Different facets of medication management were identified by these groups, ranging from having the right medication with minimal side effects, to medication adherence post‐discharge. The one service user response in this theme focused on quantity and availability, whereas researchers and HCPs focused on potential adverse events, and patient motivation to adhere to treatment in the community.

That I had enough of the right medication—service user

Be sure that the drugs are well tolerated and do not cause severe side effects—researcher

### 4.6 Symptoms/mental health

Another clinical marker often used by HCPs and researchers is symptom reduction, researchers described measuring symptom outcomes in past mental health discharge intervention research. There was agreement amongst researchers and HCPs that measuring symptoms should continue and that an effective discharge happens when the reasons for admission have been resolved *‘resolution of initial issues’* (mental health nurse). However, researchers also highlighted the problems with measuring mental health symptoms as there are no ‘biomarkers’. Service users and families had contrasting responses, only one service user described mental health, and that was in terms of wellbeing *‘how well the person feels’* (service user).

### 4.7 Service or Systems Level

The above clinical theme highlights safety on an individual, clinical level. The second theme, service/system‐level factors, address safety more widely, confronting the systems in which the care is provided. The three subthemes described within this category were (a) readmission, (b) discharge planning and (c) integration and communication.

### 4.8 Readmission

Readmission, both a clinical and systematic risk, was the second most common outcome reportedly used previously by researchers. However, one only group described readmission in relation to safety: end users of research. This group also frequently deemed an effective discharge one that *‘stops people coming back to hospital’* (service manager). For HCPs, there was an assumption that readmission within a short period of time was indicative of an ineffective discharge. Service users considered readmission differently, rather than a marker of ineffective service, service users felt readmission was sometimes something to fear, which averts help‐seeking behaviour.

not to be in fear of being put back into hospital when asking for help or support—service user.

Some service users and HCPs exemplified similar opinions about how readmission can be used as a catalyst for learning about safety and an opportunity to deliver person‐centred care. Many described the importance of exploring the reasons for readmission by assessing the patient individually ‘*exploring causes for readmission’* (mental health nurse), rather than measuring frequency of occurrence.

### 4.9 Discharge planning

Discharge planning was a key thematic subtheme across the responses of service users and families. The implications of an inconsistent, difficult to access, fragmented support system were described in detail, particularly when service users were asked to describe the most difficult elements of their discharge. Not understanding or knowing what support is available post‐discharge was considered a safety threat by many.

Inconsistency of post‐discharge support…you see a whole load of different practitioners who invariably haven't read your notes and don't know your case history—service user.

Not knowing what support will be available post discharge—service user.

Another prominent theme throughout the service user and carer responses was the safety implications of not being involved in discharge planning or shared decision‐making. *‘Not being listened to when it a bad idea ‐ then being blamed when the person relapses and ends up back again’*. Many service users feel that being involved in shared decision‐making was imperative *‘clear and precise care plan family and users involved in decision making process before discharge.’*

When service users were asked to suggest improvements to discharge procedures, many focused on improved discharge planning, follow‐up and community support. Availability of services and continuity of care, either through follow‐up plans or assigned/named members of staff were also important. For service users, safety often related to increased or more effective support throughout the transition.

Continued intensive follow‐up in the community with the same assigned staff member for the following fortnight—service user

Follow up plan with consistent support between hospital and community‐ named professional.—researcher

### 4.10 Integration and communication between services

There was agreement between groups that better integration of services would improve safety at discharge and that a fragmented care system can be dangerous. The communication between secondary care professionals and primary/community/social care professionals was described considerably in the responses.

integration between the different roles of health and social care—health services manager.

ward team and community team working closely together—service user.

### 4.11 Human, behavioural and social elements of safe transitions

This overarching category focused on the individual, psychological and social elements of safety described by the participants. This consisted of (a) readiness for discharge, (b) social networks/support, (c) adaptation to normality, (d) isolation/loneliness and (e) knowledge.

### 4.12 Readiness for discharge

The term ‘readiness for discharge’ was highlighted throughout; however, the manner of articulation varied. For example, researchers discussed *‘preparation for discharge’*, a passive process done to the service user, HCPs often described readiness from a clinical perspective *‘feeling well enough’*, whereas service users focused on being emotionally and psychologically ready for discharge *‘feeling safe to leave, as the ward may have been a place of safety’*. Family members also described their own readiness as a safety issue *‘not being included in the discharge planning and you are not ready for the person to return home’*.

Service users, HCPs and managers all acknowledged that the increasing systems‐level pressure for beds, means many service users are discharged earlier. One service user describes ‘not being ready to leave but not having a choice whether to be discharged or not’, which is echoed by nurses on the acute ward ‘often we are finding that they do not want to be discharged’. A clinical psychologist uses the analogy of a sticking plaster:

Ensuring people are not just discharged when they are safe enough and readmitted when they deteriorate‐ it can feel like a sticking plaster

### 4.13 Social networks and support systems during transitions

A social network or formal/professional support system is considered key by all groups. However, there is again disparity in how the groups conceptualize this, for service users/families it generally concerned professional or formal support in the community *‘‘support services in the community’* (service user). Contrastingly, HCPs and researchers focused on the importance of informal support *‘Connection to brighter social system’* (clinical psychologist). There is underlying consensus in the responses from researchers and end users of research that informal social support can increase safety: *‘opportunities to build support networks which could serve as protective factors and prevent readmission’* (researcher). Researchers and HCPs also highlight *‘social networks’* as a future priority, but this perspective was not shared by service users.

### 4.14 Adaptation to Normality after Discharge

The responses indicated that all groups recognized the potentially distressing transition from acute hospitalization to *‘normality’* (whatever that may be). This theme is present across responses of all other groups, but rarely described by researchers, this may be because it is captured using other terminology (eg functioning/meaningful activity). The responses exemplify differing definitions of ‘normality’. Service users sometimes described feelings of intense unsafety after immediate cessation of care, followed by an expectation to be independent. Service users associated the cessation of care with a loss of feelings of safety. Yet generally, most service users and families/carers conceptualized ‘normality’ in a positive light, a challenging but achievable ideal to work towards (focusing on employment or independence).

It is quite an adjustment to go from feeling very safe and supported on the ward, to suddenly having to remember and feel able to do basic things… using money again etc—service user.

Contrastingly, HCPs and service managers described different threats to safety that occur in ‘normality’, highlighting the effects of retuning to a problematic environment with negative implications. The language used by some HCPs highlighted an implicit assumption that a negative environment is inevitable for most:

Service users going back into the same environment that may have caused them to be become unwell in the first place ‐ where the environment has not changed or the person has not developed addition skills to cope (health service manager).

### 4.15 Isolation and loneliness

Both service users and HCPs stated that isolation and loneliness is the most difficult part of discharge from acute services. Service users described this in the context of their own emotions *‘Loneliness alone at home after the busyness of hospital ward’* (service user), whereas HCPs described this in terms of limited contact with professional services or support *‘Being isolated in the community without easy access to support at home*’(nurse). Contrastingly, no carers and family members described loneliness of service users, but instead a number described their own feelings of loneliness when they regained responsibility *‘Feeling alone with the responsibility of caring for someone who is still very unwell and perhaps suicidal’*.

### 4.16 Service user knowledge

Whether or not service users understand their condition and the healthcare systems navigated was a major safety concern for numerous groups. A lack of knowledge about condition, services and processes was described as one of the most difficult/dangerous elements by service users and their carers *‘one of the most difficult elements is a lack of psychoeducation’*. Service users continually described how they would feel safer with more knowledge *‘A clear plan and understanding of what will happen and how to access support if necessary’*. Carers and researchers alike, felt that understanding one's own condition after discharge, could be used as an indicator for improvement *‘I have hoped for more insight into his own condition’* (family member).

## 5 DISCUSSION

The main finding of this study is that safety in mental health discharge is perceived differently by service users and families compared to HCPs and researchers. Traditional safety indicators such as suicide, self‐harm, risk or adverse drug events are raised as important safety outcomes and priorities, particularly by HCPs. However, service users and families in particular have a much wider perception of safety outcomes and priorities in mental health discharge, which incorporates human, behavioural and social and system‐level factors. It could be argued that this disparity reflects a generally regimented policy‐driven approach to safety for professionals. Contrastingly, service users/family consider safety largely non‐clinically, focusing instead on human elements of communication, emotions and relationships. The regimented, outcome‐based, checklist approach has improved safety in many clinical environments and is particularly useful in reducing surgical errors or controlling infection.^24^ However, in the context of mental health services, this study highlights the importance of widening perceptions of patient safety because a mental health population has distinct differences to other clinical populations; many of the illnesses do not have physical indicators. There is a drive in healthcare practice and research to substantially involve those with lived experience in the design of research projects and quality improvement,^19^, ^25^ this study exemplifies this.

When service users were asked about safety they rarely described risky components of their own behaviour (ie ‘self‐harm’, ‘violence’, ‘aggression’ or ‘medication non‐adherence’), but instead consistently focused on the elements of the system/service that could be improved. For example: shared decision‐making, appropriate accommodation, being satisfied with information provision and service availability. Shared decision‐making is recognized as a guiding principle of mental health policy and practice, and yet despite a sustained policy emphasis, this has yet to be effectively translated into practice.^26^, ^27^ For this reason, researchers commonly considered it an example of good practice (or a proxy variable) instead of an outcome to indicate intervention effectiveness.^20^ Most groups felt safety would be improved if service users are involved in decision‐making around their transitional care planning. This research adds to the growing body of literature in this field advocating for shared decision‐making to be translated from policy to practice.^25^, ^26^, ^27^

The safety implications of ‘readiness for discharge’ are described considerably, with differing definitions, service users described not feeling emotionally ready, as opposed to clinical readiness highlighted by HCPs. Recent research outlines the lack of consensus definition on readiness for hospital discharge, which is characterized by four distinct elements: physical stability/home self‐management; support to cope after discharge; psychological ability to manage the process; and adequate information/knowledge to respond to common problems.^28^ Our research highlights how all components of the definition are present and arguably a universal understanding across groups, that ‘not being ready for discharge’, by any definition, poses threats to safety.

Our findings are congruent with past safety research that mental health safety issues concerning discharge require a widening of existing safety categorization/classification. Recently, Berzin's et al^2^ added two new categories: ‘service processes’ and ‘social environment’, to an existing framework. These categories epitomize the concerns of the service users/families in this study. This work highlights different ‘cultures of risk’ between the lived experience and professional groups, literature suggests different groups of society form their own view of an environment, which guides the risk on which they focus their attention.^29^ This work highlights the difference between the scientific‐technical cultures and the lived experience cultures of risk. Researchers and HCPs tend to perceive risk from a biomedical/technical perspective, whereby certain risks are relatively well defined in the clinical literature, measurable and to some extent definitive. Thinking of risk in this way can lead to technical practices for risk mitigation and an opportunity to define the problem by the solution. For example, considering risk of medication management, presents more opportunities for clinically evidenced solutions. For service users and their families, safety is much wider than what can be measured, they do not apply a technical lens and instead focus on the elements of day‐to‐day life that could be improved. Articulating and incorporating both views of risk have potential to underpin optimal multifaceted quality and safety interventions.

Whilst an existing body of evidence highlights the differences in perceptions between service users and mental healthcare professionals,^30^, ^31^, ^32^ the novelty of this study is that it provides a better understanding of how safety is conceptualized specifically in transitions of mental health care, a key research and policy priority for the WHO.^5^ This work also highlights the importance of ensuring the patient voice is heard in future mental transition quality and safety research, as perceptions of safety vary considerably between groups and current transitions research, policy and practice frameworks are based predominantly on the biomedical and technical perspective of healthcare professionals and researchers.

### 5.1 Strengths and limitations

This is the first study using this method to understand the differing stakeholder perceptions of patient safety in mental health discharge. However, as the service user and carer groups are predominantly UK‐based and could have been more international, this potentially limits the applicability of these results. Finally, service users and family/carers are represented equally to HCPs in this study. A lived experience expert was part of this study team and involved in research design, recruitment, analysis and manuscript preparation.

This study was opportunistic and small in scope and scale and the service user and family/carers in the sample, self‐identified using online recruitment. This was a secondary analysis of data captured for another primary purpose (eliciting potential outcomes that could be included in a core outcome set).^20^ Therefore, the relatively small sample was not sampled for a group comparison study. Numerous participants were the only participant from their country and given the diversity of the countries and the care systems within them, any results are not conclusive, for example service user involvement in decision‐making differs across countries. As in much qualitative work, theme and subtheme development could be considered arbitrary; there is potential overlap in themes as that is the nature of conducting systems research, which is often considered ‘messy’. Other thematic groupings are possible with this data set, but the ones we have chosen we feel are the best fit for our data and answering our research question.

### 5.2 Future research

Future research should focus on refining/evaluating interventions to improve discharge. Adopting a wider view of safety, including social outcomes that are rarely measured in clinical trials,^1^ could allow for researchers to be explicit in terms of outlining theory of change. Bringing multiple perspectives together to co‐design potential solutions and including patients and carers/family members is key for future quality and safety mental health transition intervention development.^33^ We foresee that improving patient safety in mental health transitions requires a tailored and multifaceted approach to target clinical, system and human, behavioural and social factors.

## 6 CONCLUSION

Patient safety in mental health‐care transitions is complex and perspectives about its critical components and priorities differ across different groups. Whilst suicide, violence, self‐harm and risk are generally considered important by most groups, there is impetus to widen definitions so that research and practice acknowledge the importance of systems, human, behavioural and social elements. Responses reveal that, unlike researchers and HCPs, service users, families and carers rarely describe clinical outcomes when considering safety at discharge, whereas systems, human, behavioural and social outcomes (such as readiness for discharge and knowledge) are their main concern, the difference in perceptions of safety provides evidence for the importance of working alongside lived experience experts in the design, conduct and analysis of quality and safety research.

## CONFLICT OF INTEREST

No authors report competing interests.

## AUTHOR CONTRIBUTIONS

NT conducted the study, analysis and drafted the majority of the manuscript. NT and JW supervised the project and contributed to the analysis and manuscript drafting. AG was the lived experience expert who advised about service user perspective throughout the project, and he also contributed to analysis and manuscript drafting. MP provided supervision for the analysis and drafting the manuscript.

## Supporting information

Table S1‐S2

## Data Availability

The data sets used and/or analysed during the current study are available from the corresponding author on reasonable request.
