# Supplementary material for: What does safety in mental healthcare transitions mean for service users and other stakeholder groups: An open‐ended questionnaire study
Source: Health Expect. 2021 Jan 20;24(Suppl 1):185–94. doi: 10.1111/hex.13190 (PMC8137494; doi:10.1111/hex.13190)
Supplement: Supplementary file 1 — Table S1‐S2 [file HEX-24-185-s001.docx]

| Group |  |
| --- | --- |
| All Participants | What would make discharge from an acute mental health ward **safe** in your opinion? |
| All Participants | What would make discharge from an acute mental health ward **effective** in your opinion? |
| All Participants | What would you like to see prioritised for research into discharge from an acute ward? |
| All Participants | Can you think of any important outcomes to measure in research assessing discharge interventions? |
| Service User | What do you think is the most difficult aspect of discharge from a mental health acute ward? |
| Service User | What might improve discharge from a mental health ward? |
| Service User | Was there any one person/group/intervention which really made a difference for you? If so what was it? What made it so powerful? |
| Service User | Do you have any other feelings or concerns not addressed in previous questions? |
| Family member/carer | What do you consider to be the most difficult components of discharge for your family member/friend from an acute mental health ward? |
| Family member/carer | What types of improvement would you expect to see in your family member/friend following a successful discharge from an acute mental health ward? |
| Mental health professional | What do you consider to be the most difficult aspects of discharge from an acute unit for your service users? |
| Mental health professional | Are there any changes/behaviours you would expect to see in a service-user during or following a safe and/or effective discharge? |
| Researchers | If applicable, what outcomes did you measure in past research (of discharge interventions)? |
| Researchers | Are there any concepts that you think are important to measure, but chose not to, due to not having a suitable measurement instrument? |
| End users of research | If you were looking to use research to inform changes to the discharge procedure within your professional role, what outcomes would you like to see reported? |
| End users of research | In your opinion, what measurements would persuade you that a discharge intervention is effective? |

**Supplementary File 1: Table of the questions presented to each group in the questionnaire**

**Supplementary File 2: Participant Demographics**

| **Country of Residence** | **Frequency/Percentage** |
| --- | --- |
| England and Wales | 69 (74%) |
| USA | 7 (8%) |
| Australia | 4 (4%) |
| Switzerland | 4 (4%) |
| Canada | 2 (2%) |
| France | 1 (1%) |
| Germany | 1 (1%) |
| Hong Kong | 1 (1%) |
| Iran | 1 (1%) |
| Italy | 1 (1%) |
| Northern Ireland | 1 (1%) |
| South Africa | 1 (1%) |

**Country of Residence**

**Gender**

| **Gender** | **Frequency/Percentage** |
| --- | --- |
| Female | 28 (30%) |
| Male | 63 (68%) |
| Not disclosed | 2 (2%) |

**Age**

| **Age** | **Frequency/Percentage** |
| --- | --- |
| 18-24 | 4 (4%) |
| 25-34 | 20 (22%) |
| 35-44 | 30 (32%) |
| 45-54 | 24 (26%) |
| 55-64 | 10 (11%) |
| 65-74 | 4 (4%) |
| 75 + | 1 (1%) |

**Stakeholder Groups (Participants chose all that applied)**

| **Group** | **Frequency/Percentage** |
| --- | --- |
| Service user/patient with lived experience | 27 (20%) |
| Family member/informal carer | 17 (13%) |
| Mental health professional | 39 (29%) |
| Researcher | 37 (27%) |
| End user of research (policy maker, mental health advocate, health services manager, charity/third sector worker) | 15 (11%) |
